# Epidemiological Characteristics of MERS-CoV Human Cases, 2012- 2025

**DOI:** 10.1007/s44197-025-00446-2

**Published:** 2025-08-06

**Authors:** Mazin Barry

**Affiliations:** 1 Infectious Disease Unit, Department of Internal Medicine, College of Medicine, King Saud University, Riyadh, Saudi Arabia; 2 Division of Infectious Diseases, Faculty of Medicine, University of Ottawa, Ottawa, Canada

**Keywords:** Middle East respiratory syndrome coronavirus, MERS-CoV, Kingdom of Saudi Arabia, Epidemiology, 2012–2025

## Abstract

**Aim:**

To describe the epidemiological characteristics of Middle East respiratory syndrome coronavirus (MERS-CoV) human cases since the first reported case in 2012.

**Methods:**

This is a retrospective descriptive epidemiological analysis of all laboratory-confirmed MERS-CoV human cases reported to the World Health Organization (WHO) from 2012 to May 2025. Cumulative cases globally, along with their demographics, comorbidities, epidemiological exposure, symptoms, hospital admissions, and mortality, were included. Descriptive analysis was used for the data.

**Results:**

Between March 2012 and May 2025, a total of 2,626 laboratory-confirmed MERS-CoV human cases were reported to the WHO, with 947 (36.1%) resulting in deaths. The majority of cases occurred in the Kingdom of Saudi Arabia (KSA), with 2,217 (84.4%) human cases and 866 (39.1%) deaths. Twenty-six other countries reported human cases, with the highest number occurring in South Korea, which reported 186 cases (7.1%). The highest number of cases occurred in 2014, with 662 (29.9%) cases, followed by 2015, with 453 (20.4%) cases. Almost half of the cases in KSA (44.7%) were secondary infections, and most (83%) required hospital admission, with 39.7% requiring admission to intensive care unit. The most common comorbidities were diabetes mellitus, chronic heart disease, and chronic renal failure. Between 2020 and the end of May 2025, 113 new human cases of MERS-CoV infection (4.3%) were reported, with the majority occurring in KSA. In 2025 alone, 10 new cases were reported, with two deaths. Secondary transmission occurred in 60% of these cases. Seven of the 10 cases were reported in April 2025 alone.

**Conclusion:**

Between 2012 and May 2025, the majority of MERS-CoV infections occurred in the Kingdom of Saudi Arabia and had a high mortality, reaching 40%. Although most cases were reported between 2014 and 2015, new human cases are still ongoing and are increasing in 2025. Continued epidemiological investigation and surveillance are needed.

## Introduction

The Middle East respiratory syndrome coronavirus (MERS-CoV), the causative agent of Middle East respiratory syndrome (MERS), first emerged in 2012 in the Kingdom of Saudi Arabia (KSA), where a novel coronavirus (HCoV-EMC) was first isolated from a middle-aged man who presented with severe pneumonia, which rapidly progressed to acute respiratory distress syndrome (ARDS), progressive renal failure and ultimately died 11 days after admission [1]. Phylogenetic analysis demonstrated that this novel virus is a betacoronavirus closely related to bat coronaviruses HKU4 and HKU5. The patient lived in Al-Bisha, a rural area in KSA. He owned many camels, and the virus was detected in bat guano in the areas around him [1]. A second case was reported in London from a patient who was transferred while in hospital from Qatar, who presented with severe pneumonia after traveling to KSA, and he also died [1]. Then, a third case was reported in 2012 in Riyadh, although he was known to have chronic kidney disease and required invasive mechanical ventilation (IMV), he was the first to survive [2]. The first evidence of human-to-human transmission occurred within a family cluster in November 2012, in which half of the infected family members died [3]. By February 2013, 15 cases in KSA were reported: all but two perished [4]. By end of May 2013, 49 confirmed cases with 26 fatalities were reported, with 37 cases in KSA and 21 deaths, two in Jordan, who both died, four in the United Kingdom (UK), of which two died, two in Germany with one death, and one case in both France and Tunisia with no deaths [3].

Between April 1 and May 23, 2013, a large hospital outbreak occurred in the Eastern province of KSA, with 23 cases and 15 deaths [5]. Multiple hospital outbreaks throughout the KSA quickly followed, causing many casualties among patients, healthcare workers (HCWs), and disruption of healthcare facilities and services [6–12]. Very quickly, more phylogenetic analysis confirmed MERS-CoV’s bat origin [13], and full-genome deep sequencing with phylogenetic analysis suggested that its human diversity resulted from multiple zoonotic events [14]. Strong evidence from geographic and temporal surveys of camels in KSA indicates that MERS-CoV has been circulating in dromedary camels since 1992 and is distributed throughout all regions of KSA, with no evidence of other infected livestock, including domestic goats and sheep [15]. In addition, nasal swabs from camels throughout KSA demonstrated indistinguishable whole-genome sequencing from that of humans [16]. In a nationwide, cross-sectional serological study conducted between December 2012 and December 2013 involving over 10,000 individuals, 1.5% had anti-MERS-CoV antibodies. Individuals working in the shepherding and slaughterhouse sectors were 15 and 23 times more likely to be seropositive, respectively [17]. One of the main hallmarks of MERS is its high case-fatality rate of almost 40%. With no specific therapy or vaccine currently available, the potential re-emergence of MERS-CoV is concerning. Nearly thirteen years after its first discovery, with another coronavirus pandemic in between, MERS-CoV infections are still reported in KSA [18]. The current study aimed to describe the epidemiological characteristics of MERS-CoV cases from 2012 to 2025.

## Methods

This is a retrospective descriptive epidemiological analysis of all human cases of MERS-CoV infections, which were laboratory-confirmed by polymerase chain reaction (PCR) and reported to the World Health Organization (WHO) between 2012 and May 2025. Data was collected from the WHO MERS-CoV dashboard, and included cumulative reported cases globally, regions of cases, demographic characteristics, comorbidities, occupation, likely exposure as determined by epidemiological investigation; primary case defined as a laboratory-confirmed MERS-CoV infection that has no direct epidemiological link to a human MERS-CoV infection, and was acquired outside of a health-care facility presumably from direct or indirect contact with the reservoir host—dromedary camels, and a secondary case was defined as a laboratory-confirmed MERS-CoV infection with a direct epidemiological link to an individual with confirmed or probable MERS-CoV infection. In addition to the presence of symptoms, hospital and intensive care unit (ICU) admissions, and mortality [19]. Descriptive analysis was employed for categorical variables, presented in the form of frequencies and percentages.

## Results

**Fig. 1:**
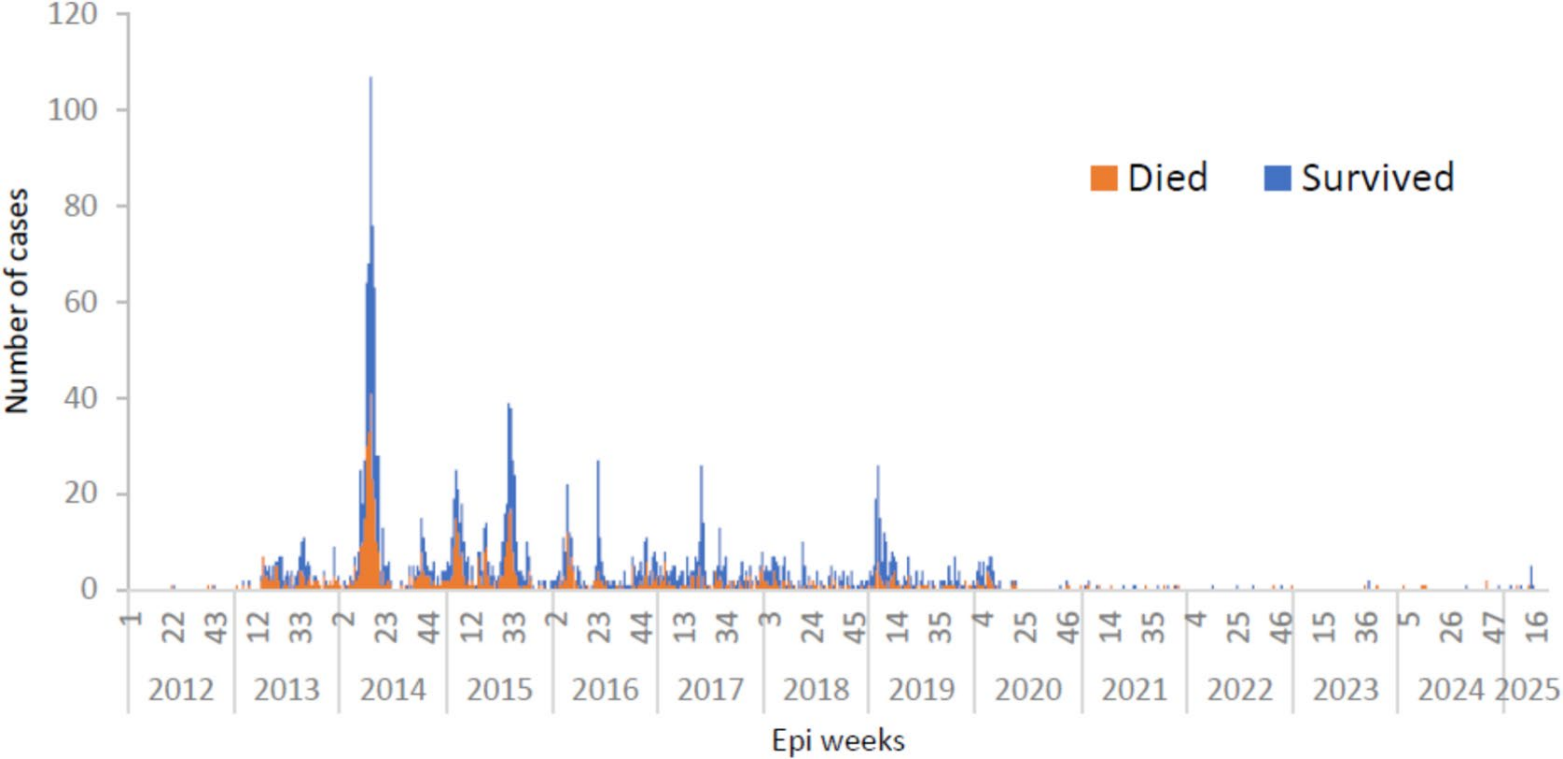
MERS cases per epidemiological week in Saudi Arabia between 2012 and May 2025. (Adapted from WHO)

Between March 25, 2012, and May 31, 2025, the cumulative number of reported human cases of laboratory-confirmed MERS-CoV infections was N = 2,626. Most cases were male, N = 1,829 (69.6%), while N = 797 (30.35%) were female. Of the total cases, N = 947 have died at a case-fatality ratio (CFR) of 36.1%. The majority of cases, N = 2,217 (84.4%), were reported from KSA with N = 866 deaths (CFR 39.1%). Globally, 26 other countries also reported human cases, including South Korea: N = 186 (7.1%), United Arab Emirates (UAE): N = 94 (3.6%), Jordan and Qatar: N = 28 (1.1%) each, Oman: N = 26 (1%), Iran: N = 6 (0.2%), United Kingdom (UK): N = 5 (0.19%), Kuwait: N = 4 (0.15%), Germany, Thailand, and Tunisia: N = 3 (0.11%) each. Two (0.08%) in Lebanon, Malaysia, the Philippines, Algeria, Austria, France, the Netherlands, and the United States of America (USA), and one (0.04%) in Yemen, Bahrain, China, Egypt, Turkey, Greece, and Italy. The highest number of cases was reported in 2014, with 662 (29.9%) human cases, the majority of which occurred between March and June 2014, accounting for 540 (20.56%) infected individuals. This was followed by 2015, which had 453 (20.4%) human cases. Most cases occurred between May and June 2015, resulting in 209 (7.96%) cases (Fig. 1).

**Fig. 2:**
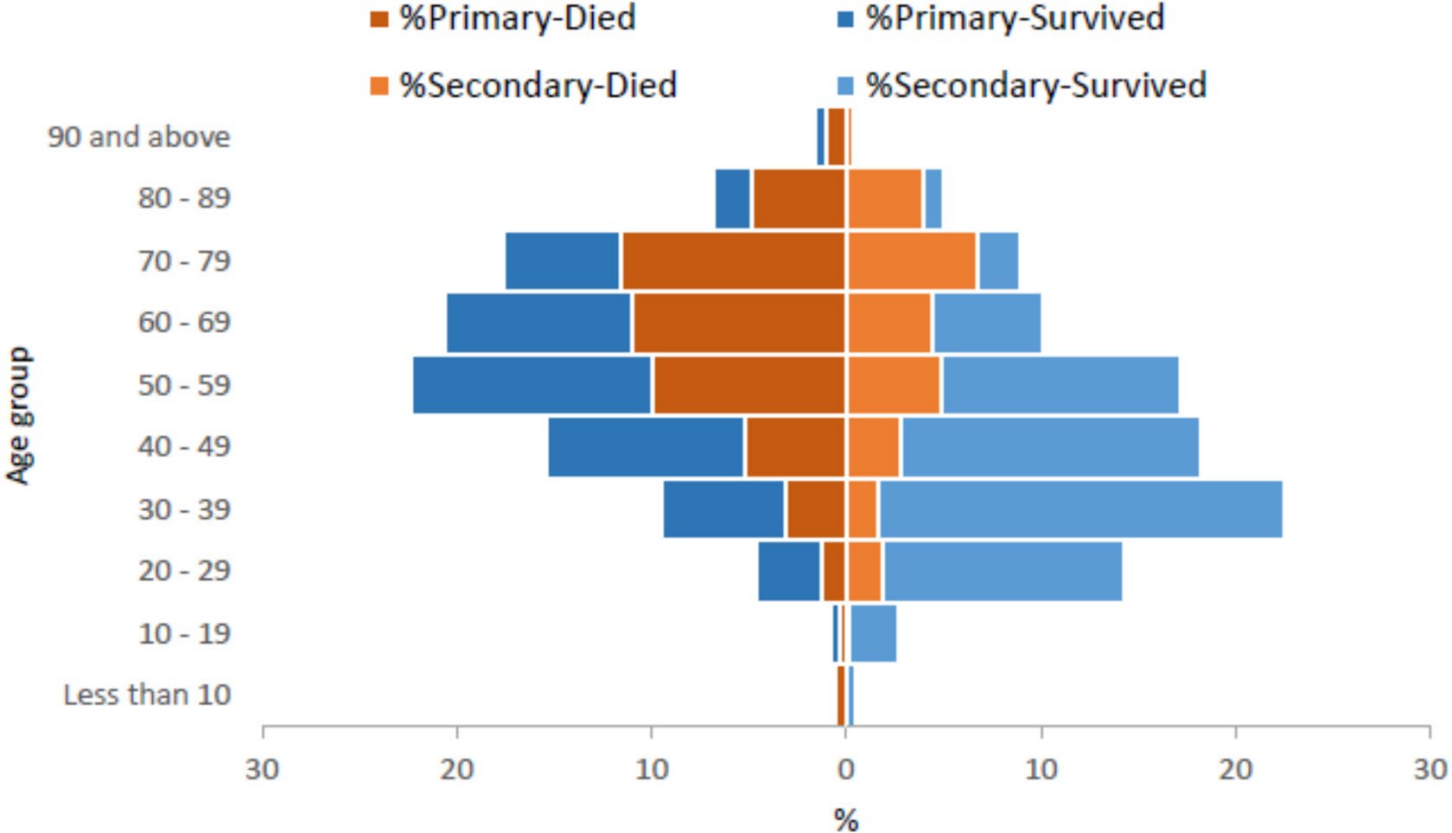
Age and mortality of primary and secondary cases of MERS in Saudi Arabia between 2012 and May 2025. (Adapted from WHO)

Of all the human cases in KSA, N = 1,225 (55.25%) were primary cases, and N = 991 (44.7%) were secondary infections. The ages varied, N = 14 (0.5%) were between 0 and 4 years old, N = 38 (1.45%) were between 5 and 17 years old, N = 1,616 (61.5%) were between 18 and 59 years old, and N = 958 (36.5%) were 60 years or older (Fig. 2).

There were N = 17 (0.64%) cases who directly worked with camels, while N = 223 (8.5%) were exposed to camels or their products. Epidemiological investigation determined that the likely place of exposure was a healthcare facility in 895 (34.1%) human cases, the community in 576 (21.9%), within the household in 227 (8.6%), and within the workplace in 5 (0.19%); however, N = 923 (35.1%) were not reported. Symptoms were reported in N = 2,177 (82.9%) of the infected individuals, while N = 176 (6.7%) remained asymptomatic. There was no data available for N = 273 (10.4%) cases regarding their symptoms. Most cases were admitted to the hospital, with N = 2,180 (83%) cases, and N = 865 (39.7%) of those admissions required ICU admission. The most common comorbidities were diabetes mellitus in N = 397 cases, chronic heart disease in N = 168 cases, chronic renal failure in N = 117 cases, hypertension in N = 72 cases, chronic pulmonary disease in N = 31 cases, and immunosuppression in N = 11 cases.

From 2020 to May 2025, a total of N = 113 cases (4.3%) have been reported, with the majority occurring in KSA. Notably, N = 10 cases were reported in 2025 alone, with one case reported in January, two in March, and seven cases in April. Two of the 10 have died (Table 1).

**Table 1:** Epidemiological characteristics for MERS cases between 2020 and May 2025

Characteristics	2020	2021	2022	2023	2024	2025
Number of cases	60	20	9	6	8	10
Median age (years)	56	59	53	73	58	52
Male gender %	82%	82%	95%	89%	67%	70%
Primary case %	83%	83%	100%	67%	83%	20%
Secondary case %	17%	17%	0%	0%	0%	60%
Unknown contact %	0%	0%	0%	33%	17%	20%
Healthcare worker %	7%	7%	0%	0%	0%	60%
Death %	38%	45%	22%	50%	75%	20%

## Discussion

The overall number of cumulative MERS cases is relatively low over the past 13 years; the vast majority of cases were reported between 2014 and 2015, accounting for 42.5% of all cases. It is vital to note that although the overall number of cases over the given timeframe is low, this has primarily been attributed to the low level of testing, even in contacts who are asymptomatic [20]. Over the past five years, more than 100 cases have been reported, with the majority occurring in 2020. However, by the end of May 2025, 10 cases had been reported. Of the latter cases, one involved indirect contact with camels, while a cluster of seven cases was reported in a single healthcare facility in Riyadh, resulting in six secondary transmissions to healthcare workers [21]. As with many previous nosocomial outbreaks in KSA, the continued occurrence of secondary cases among healthcare workers, more than a decade after the emergence of MERS and following a major pandemic, is alarming. Adherence to infection prevention and control practices has been shown to reduce and halt hospital transmission [22]. This recent outbreak underscores the importance of strict adherence to these practices.

Between 2012 and May 2025, the majority of cases (more than 84%) were reported from the Kingdom of Saudi Arabia (KSA). The case fatality ratio (CFR) was 36% overall and 39% in KSA. The high mortality rate is still observed, despite the low number of cases in 2024; 75% have perished. The high mortality rate is consistent with previous epidemiological studies in KSA that showed high mortality rates of 40%, with some rates reaching as high as 80% among infected admitted patients [10–12]. Similarly, the predominance of males in MERS cases has been well described [5, 6], which may also be related to male-dominated occupations in KSA, including slaughterhouse work and shepherding. Indeed, serological surveys have found higher seropositivity among these latter groups [17]. Furthermore, seroprevalence among dromedary camels in KSA was found to be as high as 90% [23], and camels in 86% of different regions in KSA were seropositive [24]. These camels play an essential role in spillovers to primary human cases.

South Korea had the second largest number of infected human cases, with 186 cases and 36 (19.4%) deaths that occurred in 2015 from an imported case from the Arabian Peninsula [25]. Healthcare workers have the highest occupational risk, which has been well described in numerous hospital outbreaks in KSA [10–12], as well as in South Korea, where 21% of all infected human cases were healthcare workers [26]. The high number of hospital outbreaks throughout the KSA was attributed to poor infection prevention and control infrastructure [6, 12], which led to the scaling up of these measures and a subsequent decrease in such outbreaks, in addition to an unexpected benefit in preparedness for the Coronavirus disease 2019 (COVID-19) pandemic [27]. In a comparative analysis of outbreaks of MERS in South Korea and KSA, several exacerbating factors were identified in the latter country, including poor access to healthcare professionals at general practitioner level, denial of association of MERS-CoV with camels, 75% of healthcare workers were unaware of how the disease spreads, and the general feeling by healthcare workers, as well as by citizens, that MERS was not a substantial problem [28].

The number of cases in countries outside the Arabian Peninsula, excluding South Korea, was generally low, and almost all have been linked to travel to the Middle East. This clearly illustrates the underlying epidemic potential of MERS-CoV [29]. Severe acute respiratory syndrome coronavirus (SARS-CoV) caused a world outbreak in 2002–2003, when it spread from Hong Kong to 37 different countries, infecting 8,098 human cases with 774 deaths [30], and more recently, the devastation of Severe acute respiratory syndrome coronavirus-2 (SARS-CoV-2), with the COVID-19 pandemic, that has infected almost 778 million people between December 2019 and May 2025 [31], with more than 7 million deaths, the world would be devastated with another possible epidemic which potentially can be caused by MERS-CoV.

Within the KSA, in the two consecutive years with the highest number of cases, 2014–2015, late spring seemed to have the peak incidence of infections. Some evidence suggests that the seasonality of MERS-CoV may be related to spillovers from camels to humans, which could be temporally linked to camel-related activities, including calving and weaning, that occur annually or biannually. The WHO has recommended cross-sectional studies in populations exposed to such activities [32]; the current study supports this notion, particularly given the 2025 cases that arose within a similar season.

Most reported cases had symptoms, as MERS sufferers tend to have a broad spectrum of symptoms from mild to ARDS with acute renal failure, the low number of asymptomatic cases is most likely because those with mild or no symptoms do not present to healthcare facilities, and that contact tracing in the KSA do not test for asymptomatic individuals [20, 33]. The latter cases may lead to further infections if undetected. More than one-third of the cases identified in the current study are secondary cases. This has been previously documented, with a high number of infections occurring among patients and healthcare workers, as well as between patients and visitors [34].

The number of annual cases in KSA has steadily dropped since 2020, which coincided with the emergence of the COVID-19 pandemic. Only eight cases have been reported with co-infection of the two coronaviruses, with three deaths [35]; however, the overall reported cases remained unexpectedly low. Possible reasons for this include the overwhelming impact of the COVID-19 pandemic, which compromised the surveillance of MERS by prioritizing SARS-CoV-2 over MERS-CoV despite their similar clinical presentation [36]. The recent cases in 2025, with ongoing secondary cases and the high mortality rate, highlight the ongoing threat posed by MERS-CoV and its potential to cause explosive epidemics in the KSA and beyond.

The current study has several limitations; the data analyzed are purely descriptive of human laboratory-confirmed cases to the WHO, which would miss many suspected cases that have not been confirmed or those that were not reported. Some data were not thoroughly documented, including the type of exposure and the presence of symptoms, as well as a lack of details on symptomatology.

The current study provides a brief description of the epidemiological characteristics of MERS-CoV human cases from its initial emergence in 2012 to the end of May 2025, highlighting various aspects and features of the disease, as well as gaps in current knowledge due to the ongoing spread of MERS-CoV and its potential to cause further outbreaks. Many questions regarding the epidemiology of MERS-CoV, particularly within KSA, remain unanswered. Including what are the possible modes of transmission for MERS-CoV and associated transmission-based precautions that have been previously recommended and successful, and what is the duration of the infectious period of MERS-CoV? Such questions and their answers require continued active surveillance, comprehensive epidemiological investigations, and continued reporting. Further epidemiological studies are warranted to help answer these questions.

## Data Availability

No datasets were generated or analysed during the current study.
